# Hydrophobic Janus Foam Motors: Self-Propulsion and On-The-Fly Oil Absorption

**DOI:** 10.3390/mi9010023

**Published:** 2018-01-11

**Authors:** Xiaofeng Li, Fangzhi Mou, Jingjing Guo, Zhuoyi Deng, Chuanrui Chen, Leilei Xu, Ming Luo, Jianguo Guan

**Affiliations:** State Key Laboratory of Advanced Technology for Materials Synthesis and Processing, International School of Materials Science and Engineering, Wuhan University of Technology, Wuhan 430070, China; xiaofegli@whut.edu.cn (X.L.); october@whut.edu.cn (J.G.); zydeng2017@whut.edu.cn (Z.D.); chc034@ucsd.edu (C.C.); xull@whut.edu.cn (L.X.); luoming_2016@whut.edu.cn (M.L.)

**Keywords:** self-propulsion, oil/water separation, foam, capillary interaction, Marangoni effect

## Abstract

In this work, we for the first time have proposed and fabricated a self-propelled Janus foam motor for on-the-fly oil absorption on water by simply loading camphor/stearic acid (SA) mixture as fuels into one end of the SA-modified polyvinyl alcohol (PVA) foam. The as-fabricated Janus foam motors show an efficient Marangoni effect-based self-propulsion on water for a long lifetime due to the effective inhibition of the rapid release of camphor by the hydrophobic SA in the fuel mixture. Furthermore, they can automatically search, capture, and absorb oil droplets on the fly, and then be spontaneously self-assembled after oil absorption due to the self-propulsion of the motors as well as the attractive capillary interactions between the motors and oil droplets. This facilitates the subsequent collection of the motors from water after the treatment. Since the as-developed Janus foam motors can effectively integrate intriguing behaviors of the self-propulsion, efficient oil capture, and spontaneous self-assembly, they hold great promise for practical applications in water treatment.

## 1. Introduction

Oil-leakages/spillages from industrial oily wastes or ship accidents bring many toxic compounds to water resources and may cause disastrous consequences to public health and aquatic ecosystems [1,2,3,4]. To address these issues, growing efforts have been devoted to the development of new materials for oil absorption and oil/water separation. The practical application of these materials requires them to have a high oil/water separation efficiency and low cost. Conventional low-cost materials for oil removal, including clay, activated carbon, and natural fibrous sorbent usually bring secondary pollutants into water and suffer from low selectivity, low absorption capacity, and long separation times [5,6,7,8]. In recent years, superhydrophobic and superoleophilic meshes, membranes, fabrics, nanofibers, etc. have been developed and used as filters for selective oil separation [4,9,10,11,12]. Even though they have been recognized to effectively separate oil contaminants from water, they are less practical on oil spills on open water because the contaminated water needs to be collected first for subsequent filtration [13,14,15,16].

Commercial sponge and foam are cheap porous materials and are available in our daily lives, and their interconnected 3D skeleton structures are expected to endow them with huge spaces for oil absorption and storage [17]. In fact, without surface modification, the pristine foam/sponge materials have a poor selectivity and may absorb both oil and water. Enhancing the roughness of foam surfaces and reducing foam surface energy are two common strategies to modify foam for the selective oil absorption from polluted water [18], which include methods such as dip coating [19], in-situ chemical reactions [20], vapor deposition [21], and spray coating [22]. Compared with other methods, dip coating is an effective and low-cost method for depositing hydrophobic materials onto substrates. After surface modification, the sponge and foam-based oil/water separation materials are promising for efficient oil/water separation with obvious advantages, such as high oil absorption capacity, low cost, and high selectivity [15]. However, those foams still cannot be directly used to deal with oil contaminants on open water owing to a lack of strategies to manipulate foams to capture oil floating on water. In particular, recent studies have demonstrated that magnetic foams, which are fabricated by incorporating magnetic particles onto porous foams, can be manipulated under an external magnetic field to target specific oil contaminants on water [23,24,25]. However, the incorporation of magnetic particles on porous foam complicates the fabrication process and raises the cost of the foams. In addition, as magnetic flux density decreases intensively with distance, the magnetically targeted oil removal is hindered in wide-range water treatment.

Self-propelled motors/objects have attracted considerable attentions over the last decade due to their conversion of other forms of energy into autonomous motion [26,27,28,29]. They are capable of picking up, transporting, and releasing various cargoes and thus have shown considerable promises for environmental detection and remediation, such as oil removal [30,31,32]. For instance, by modifying with hydrophobic organic chains on the surface, self-propelled micromotors, and microengines are capable of capturing oil droplets in water [33,34]. However, to power these micromotors and microengines, surfactants or chemical fuel (e.g., H_2_O_2_) need to be introduced into the aquatic environment, which not only makes the oil separation less cost-effective but also brings secondary pollutants into the environment. In addition, these micromotors and microengines can only capture the suspended oil droplets in water and are less effective to deal with the floating oil contaminants from oil spill and industrial oily wastewater. Alternatively, the self-propelled polysulfone (PSf) capsules loaded with sodium dodecyl sulfate (SDS) have been proposed to repel floating oil droplets through Marangoni effect, and realize the collision and merging of the scattered oil droplets [32]. However, it is difficult for the capsules to gather oil droplets on wide-open water due to the limited distance (several centimeters) of the repulsive interaction. In addition, as they can only translocate or merge oil droplets on water, an additional process is needed to separate oil from contaminated water. Hence, an ideal self-propelled motor for oil absorption is expected to have the capabilities of capturing and realizing in-situ oil/water separation in wide-open water, thus enhancing the overall performance and avoiding excessive redundancy.

In this work, we have proposed a self-propelled Janus foam motor, which is fabricated by simply loading the camphor/stearic acid (SA) mixture as fuels into one end of the SA-modified polyvinyl alcohol (PVA) foam, for on-the-fly oil absorption on water. The Janus foam motor shows an efficient self-propulsion on water with a long lifetime based on Marangoni effect owing to the sustained asymmetric release of camphor. Due to the attractive capillary interactions between motor and oil droplets and that between motors, the Janus foam motors not only can effectively capture and absorb oil droplets on the fly but also exhibit spontaneous self-assembly after the oil absorption, which facilitates the subsequent collection of the Janus foam motors from water after the treatment. Compared to the immobile or magnetically-driven hydrophobic foams [15,23,24,25], the as-developed Janus foam motors are expected to exhibit a much higher efficiency for oil removal because they can automatically search and capture oil contaminations in a wide range and then self-assemble into aggregates after oil absorption, due to their integrated properties of self-propulsion and hydrophobicity.

## 2. Materials and Methods

### 2.1. Fabrication of Janus Foam Motors

Commercial PVA foam was firstly cut into bar-shaped pieces with dimensions of 10 mm × 3 mm × 3 mm (length, width, thickness). The bar-shaped PVA foam (10 mg) was immersed into an ethanol solution with 0.35 mM stearic acid for 3 s and dried in air at room temperature to obtain SA-modified PVA foam. Afterwards, SA (29 mg) and camphor (14.5 mg) were loaded in one end of the SA-modified PVA foam by immersing it into liquid camphor/SA mixture at 80 °C. After cooling in air at room temperature, the Janus foam motor is obtained. The motors with different camphor mass ratio (*r_c_*) were also fabricated using the same method to investigate the influence of *r_c_* on the speed and lifetime of the motors. To adjust *r_c_*, the weight of camphor was varied in the mixture while keeping SA weight (29 mg) unchanged. The Janus foam motor loaded with pure camphor was prepared by replacing 29 mg of SA into 30 μL of ethanol with other conditions unchanged.

### 2.2. Characterization of Janus Foam Motors

Scanning electron microscopy (SEM) images were obtained by a Hitachi S-4800 field-emission SEM (Hitachi, Tokyo, Japan). Fourier-transformed infrared (FTIR) spectra were obtained using a Nicolet 6700 FTIR spectrometer (Thermo Fisher Scientific, Waltham, MA, USA) in the range of 400–4000 cm^−1^ with a resolution of 4 cm^−1^.

### 2.3. Self-Propulsion and Oil Absorption

Janus foam motors were placed on water in a Petri dish (diameter: 150 mm, depth: 30 mm) with 100 mL water. A high-definition (HD) video recorder was placed over the dish. The video clips were analyzed with Video Spot Tracker V8.0 software (Center for Computer Integrated Systems for Microscopy and Manipulation (CISMM), UNC Chapel Hill, NC, USA).

The paraffin oil was colored with Solvent Blue 14 (Alfa Aesar, Haverhill, MA, USA) for easy observation. Several oil droplets (10 μL) were placed on the surface of the water. Then Janus foam motors were put onto water to capture and absorb oil droplets on the fly. The oil absorption capacity (*Q*) is determined by weighing the motors before and after oil absorption, and is calculated as follows.

$$Q=\frac{\left(m_{1}-m_{0}\right)}{m_{0}}$$

Here, *m*_0_ and *m*_1_ are the weights of the motors before and after oil absorption, respectively.

## 3. Results and Discussion

**Figure 1:**
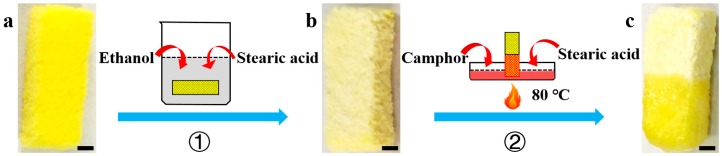
Schematic illustration of the fabrication of the Janus foam motor. At first, the pristine polyvinyl alcohol (PVA) foam (**a**) was modified with stearic acid (SA) by soaking it in SA ethanol solution (step 1) and drying in air to prepare SA-PVA foam (**b**). Then, the Janus foam motor (**c**) was prepared by loading camphor/SA mixture into one end of the SA-PVA foam at 80 °C (step 2). Scale bars: 1 mm.

The preparation of the Janus foam motor is shown in Figure 1. At first, a commercially available bar-shaped PVA foam was modified with stearic acid by a facile dipping-coating method. The pristine PVA foam (Figure 1a) shows an interconnected 3D skeleton structure, so it can quickly absorb the stearic acid solution (0.35 mM in ethanol) after dipping in the solution. The stearic acid modified PVA (SA-PVA) foam (Figure 1b) was then obtained after pulling up the foam and drying in air at room temperature to remove the solvent. Secondly, one end of SA-PVA foam was loaded with camphor/SA mixture (camphor mass ratio, *r_c_* = 0.38) by dipping it into liquid camphor/SA mixture at 80 °C. After cooling in air at room temperature, the Janus foam motor was obtained. As shown in Figure 1c, the Janus structure of the foam motor can be clearly observed, in which the upper end (SA end) of the PVA foam in light yellow color is coated with SA, and the lower end (Camphor/SA end) in bright yellow color is loaded with camphor/SA mixture. From close SEM observation, it can be seen that the pristine PVA foam before modification shows a bulk 3D macroporous structures, and the size of the pores is in several hundred micrometers (Figure S1a). After the modification, the macroporous structure remains in the SA end (Figure S1b), while the pores in the other end is completely filled by the camphor/SA mixture (Figure S1c). The uptake of camphor and SA in the Janus foam motor was confirmed by FTIR spectra of the PVA foams before and after the modification (Figure S1d). The characteristic absorption peak at 1073 cm^−1^, which corresponds to the C=O group in camphor and stearic acid [35], was observed in the PVA foams modified with SA or with camphor/SA mixture, while this peak was absent in the FTIR spectrum of pristine PVA foam.

**Figure 2:**
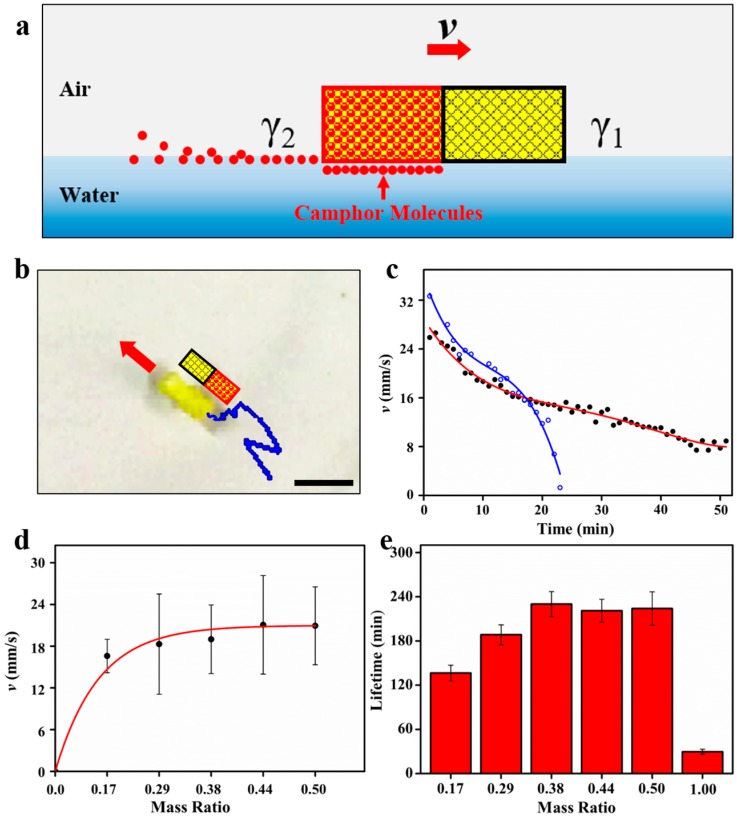
(**a**) Schematic illustration of the propulsion of the Janus foam motor based on Marangoni effect due to the asymmetric release of camphor. The red domain represents the camphor/SA end of the Janus foam motor. (**b**) Motion trajectory of the motor in 1 s. Scale bar: 10 mm. (**c**) The time-dependent speed (*v*) of the Janus foam motor loaded with the camphor/SA mixture (*r_c_* = 0.38) (black dot) and pure camphor (blue circles), respectively. (**d**) The speed (*v*) of the motor versus mass ratio of camphor in the camphor/SA mixture. (**e**) Lifetime of the motor versus mass ratio of camphor.

When the Janus foam motor is put on water, the loaded camphor can be asymmetrically released from the motor. The released camphor is then mainly dissolved in water and adsorbed at the air–water interface with a small mass loss due to its sublimation into the bulk air phase. The dissolved camphor molecules would reduce surface tension (*γ*) of water around the camphor/SA end of the motor and give rise to a gradient in water surface tension (∇γ), namely a Marangoni stress, across the motor. The Marangoni stress then propels the motor with the SA end forward [36]. The detailed propulsion mechanism of the Janus foam motor based on Marangoni effect is given in Figure 2a. The propulsion of the Janus foam motor under ∇γ can be expressed by the classical Newtonian equation [37]:

(1) $$\rho {\ddot{x}}_{c}\left(t\right)=\nabla \gamma \left(u\left(x_{c}\left(t\right),t\right)\right)-\mu \dot{x_{c}}\left(t\right)$$

where $x_{c}\left(t\right)$ denotes the center of mass of the foam motor, u(x,t) is the concentration of the diffused camphor layer, *ρ* is the surface density of the camphor, and *μ* is the surface viscosity constant. Figure 2b and Video S1 show the self-propulsion of a typical Janus foam motor on water in a Petri dish (diameter: 15 cm, depth: 3.0 cm) filled with 100 mL of deionized water. It can be seen that the motor can float on water and autonomously move with an initial speed of 24.3 mm/s. The superposition of the translational and rotational motions is frequently observed for the Janus foam motors. This can be explained by the fact that the release of camphor misalign with the horizontal axis symmetry of the motor, generating asymmetric Marangoni stresses and thus a torque rotating the motor [38]. Even though the speed of the Janus foam motor (*r_c_* = 0.38) decreased over time, a notable speed of 8.3 mm/s still remained even after 50 min (Figure 2c), indicating the continuous and sustained release of camphor fuel. In contrast, the speed of the Janus foam motor loaded with pure camphor decreased sharply from 32.8 to 0.1 mm/s in 24 min (Figure 2c), revealing the quick exhaustion of camphor fuel. From Equation (1), the propulsion of the Janus foam motor is directly related to the distribution of camphor across the motor. Thus, increasing the mass ratio of camphor in the camphor/SA end would amplify the asymmetric distribution of camphor and enhance the propulsion of the motor. As shown in Figure 2d, the speed of the Janus foam motor increases from 16.6 to 19.0 mm/s with the increasing mass ratio (*r_c_*) of the camphor from 0.17 to 0.38 and becomes stable as *r_c_* is over 0.38. The stable speed can be attributed to the fact that the surface-tension difference between the front SA end and the rear camphor/SA end becomes stable when *r_c_* is over 0.38 due to the saturated adsorption of camphor molecules at the water–air interface [39].

The lifetime of the self-propelled motors is of great significance to its various applications, such as cargo transportation, water treatment, and sensing. [40,41,42,43,44]. In this work, we elaborately loaded the camphor fuel with stearic acid, which could greatly modulate the release behavior of camphor in water due to its hydrophobic nature. In this way, rather than the quick release of the pure camphor in water due to its water solubility (1.2 g/L), camphor in the camphor/SA mixture tended to be sustainably released in a long period, endowing the Janus foam motor with a long lifetime. As shown in Figure 2e, as *r_c_* increased from 0.17 to 0.38, the lifetime of the Janus foam motor increased from 136 to 230 min because of the increasing amount of loaded camphor fuel, but it remained unchanged during further increases in the amount of the loaded camphor fuel as *r_c_* increased from 0.38 to 0.5. This implies that SA can inhibit the rapid release of camphor and elongate the lifetime of the motor at a given amount of the loaded camphor fuel. In contrast, the Janus foam motor loaded with pure camphor only showed a short lifetime of 30 min (Figure 2e). Furthermore, the lifetime of the as-developed Janus foam motor was much longer than other motors also based on the Marangoni effect. For instance, the liquid motor consisting of polyvinyl chloride (PVC) dimethylformamide (DMF) solution only shows a maximum lifetime of 23 min due to the quick exhaustion of DMF fuel [45]. From Figure 2d,e, we can conclude that the optimal *r_c_* for the Janus foam motor is 0.38, with which it exhibits a maximum speed of 19.0 mm/s and simultaneously a maximum lifetime of 230 min. Thanks to its excellent motion behaviors, the Janus foam motor can cruise on almost the entire surface of water in the Petri dish (Figure S2a), while the SA-PVA foam without camphor (Figure S2b) shows a negligible motion distance in the same period of time.

The hydrophobicity and oleophilicity of the Janus foam motors is crucial for their performance in oil capture and absorption on water surface. The contact angles (*θ*) of water on the SA end of the Janus foam motor is measured to be 138° (Figure S3), verifying its hydrophobicity. As shown in Figure S4a–c, when a drop of paraffin oil–water mixture was dropped onto the SA end of the motor, the oil was quickly absorbed by the foam, leaving only water on its surface. The oil–water separation can be explained by the fact that paraffin oil (33.0 mN/m) [46] and stearic acid (24 mN/m) [47] have a similar surface energy, and paraffin oil can easily wet the surface of SA-PVA foam, while water is strongly repelled due to its high surface tension (72.7 mN/m) [48].

**Figure 3:**
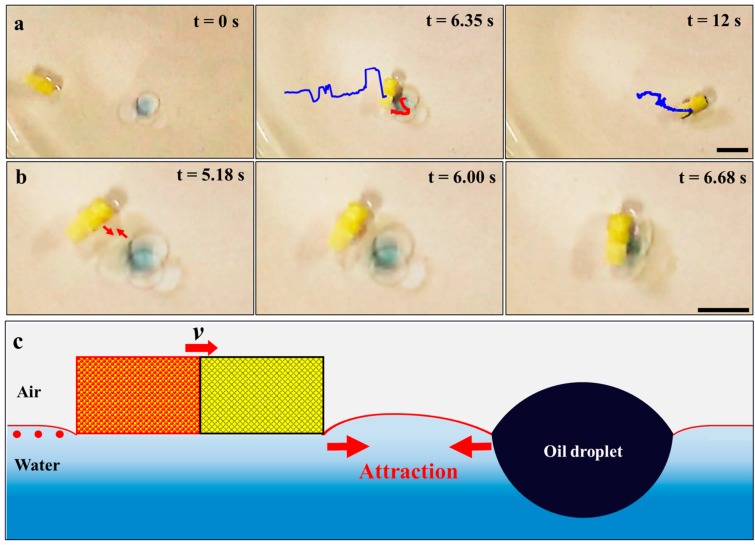
(**a**) Time-lapse images of the oil capture by the Janus foam motor. Red and blue curves represent the trajectories of the oil droplet and Janus foam motors, respectively, suggesting the autonomously approaching, capturing, and transportation of oil droplets by the Janus foam motor. (**b**) Close observation of the attraction between the Janus foam motor and an oil droplet. (**c**) Schematic demonstration of the attractive capillary interaction between the Janus foam motor and oil droplet. Scale bars: 10 mm.

Combining the motion behavior and the oleophilicity of the SA end, the Janus foam motor can perform an “on-the-fly” collection of oil on water surfaces through capillary interaction between the motor and oil. As illustrated in Figure 3a and Video S2, with the efficient self-propulsion on water, the Janus foam motor could autonomously approach (0 s), capture (6.35 s), and transport (12 s) the oil droplet (paraffin oil) on the water surface, showing an oil absorption capacity of 0.6 g/g. It is worth noting that, when the motor moves to the area in the vicinity of the oil droplet (typically in 9 mm), the immobile oil droplet would move towards the motor (red trajectory in Figure 3a,b) and then be absorbed, indicating the obvious attractive interaction between the motor and the oil droplet. Due to the hydrophobic nature of the oil droplet and the Janus foam motor, they create negative menisci at the water–air interface [49,50,51]. The formation of menisci increases the interfacial area and raises the energy of the system [52]. When these two hydrophobic objects approach each other closely enough, the menisci of the two objects overlap and interact, leading to the attractive interaction between them [51]. Then, the motor and oil droplet move toward each other spontaneously on the basis of the minimization of the interfacial free energy of the liquid–air interface [53,54]. Figure 3c shows the detailed mechanism of the attractive interaction between the self-propelled Janus foam motor and the oil droplet. The attractive interaction between the Janus foam motor and the oil droplets facilitates the motor for oil capture and absorption.

**Figure 4:**
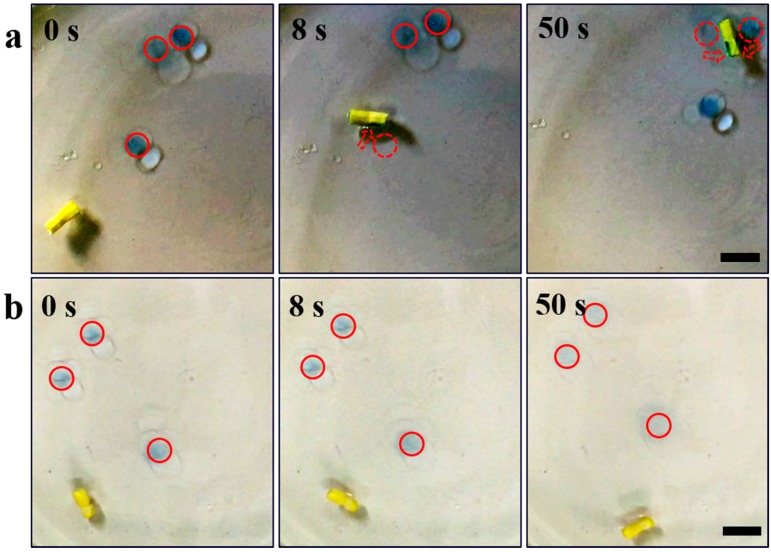
The capture of oil droplets by (**a**) a Janus foam motor and (**b**) an immobile SA-PVA foam at 0, 8, and 50 s, respectively, revealing that the Janus foam motor exhibits a much higher oil absorption efficiency than that of the immobile foam. Scale bars: 10 mm.

Even though the passive foam materials have demonstrated high removal efficiency and capacity towards the continuous oil layer or film on water [13], it is difficult to remove scattered oil droplets in a wide area as they are immobile if no external driving force is applied. As verified by Figure S2, the Janus foam motor can cruise on a wide water surface and has a much wider treating range than the immobile SA-PVA foam in the same period of time (10 min). Hence, the Janus foam motors developed in this work could capture and absorb scattered oil droplets in a wide area by self-propulsion. As shown in Figure 4 and Video S3, the Janus foam motor can effectively capture and absorb three scattered oil droplets on the water surface in 50 s (Figure 4a), while the immobile SA-PVA foam fails to capture any oil droplets (Figure 4d) because the distance between the oil droplet and the foam is beyond the capillary length (about several millimeters). These different behaviors of oil capture reveal that the Janus foam motor exhibits a much higher oil absorption efficiency than that of the immobile foam materials. Several groups have developed magnetic foams for directed oil removal [23,24,25]. These magnetic foams can be guided by external magnetic field to target specific oil contaminants on water. However, the incorporation of magnetic particles on porous foam complicates the fabrication process and raises the cost of the foams. In addition, as magnetic flux density decreases intensively with distance, the magnetically targeted oil removal is hindered in wide-range water treatment.

**Figure 5:**
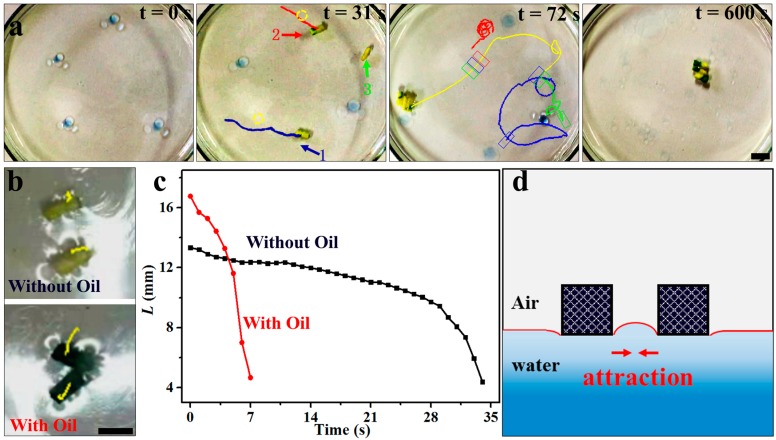
(**a**) Self-assembly of the Janus foam motors. (**b**) Trajectories of SA-PVA foams without oil and those with absorbed oil, reflecting a much faster self-assembly for the motors after oil absorption. (**c**) The distance (L) between the foams versus time. (**d**) Schematic demonstration of the self-assembly of the motors under capillary interactions. Scale bars: 10 mm.

The collection of the oil absorbers after the oil absorption is an important process of post treatment in oil remediation. It has been reported that magnetic oil absorbers, such as magnetic superhydrophobic/superoleophilic particles and magnetic foams, can be effectively collected from the treated water by magnetic separation [23]. However, the magnetic separation of oil absorbers on open water is a high energy-consuming process and can only be applied in a short range, so it may be limited in large-scale application. Alternatively, the as-developed Janus foam motors exhibit a spontaneous self-assembly behavior, facilitating their subsequent collection and separation after the oil absorption, as shown in Figure 5a and Video S4. At first, Janus foam motors 1 and 2 cruise on water for oil collecting separately. They then merge into a dimer where they collide with each other. The formed dimer can continuously move on water and grasp the Janus foam motor 3 in its path, forming into a trimer. It is reasonable to speculate that the moving trimer is able to further collect additional motors when they emerge in its motion path and finally grow into a large aggregate. The Janus foam motors with the absorbed oil are expected to show a much faster assembly than those without oil absorption, as evidenced by the results shown in Figure 5b,c and Video S5. It can be seen that two oil-absorbed foams with a distance of 17 mm could merge into a dimer in 7 s, while two oil-free foams (13 mm in distance) take over 30 s to assemble. The enhanced assembly of the oil-absorbed foams is ascribed to the elongated interaction distance between the motors owing to the diffusive spreading of the captured oil on water [55]. The spontaneous assembly of the motors is attributed to the attractive capillary interactions between them, as shown in Figure 5d. For the practical application of the Janus form motor in water treatment, it should be noted that camphor, which is toxic in large doses, does not accumulate in the environment due to their ready metabolism by many bacteria [56,57,58].

## 4. Conclusions

In summary, we have demonstrated a novel design of a Janus foam motor and its capability for on-the-fly oil absorption. The Janus foam motor was fabricated by simply loading camphor/SA mixture into one end of the SA-modified PVA foam. It demonstrated efficient self-propulsion on water with a maximum speed of 24.3 mm/s and a long lifetime up to 230 min. The self-propulsion of the Janus foam motor stemmed from the sustained asymmetric release of camphor from the motor, generating Marangoni propulsion. Due to the hydrophobic nature of the surface of the Janus foam motors, they not only can automatically search, capture, and absorb oil droplets on the fly but also can spontaneously merge into large aggregates on water after the oil absorption under the attractive capillary interactions between them. As the as-developed Janus foam motors effectively integrate three intriguing behaviors, including self-propulsion, efficient oil capture, and the spontaneous self-assembly, they may hold great promise for applications in water treatment.

## Supplementary Materials

The following are available online at www.mdpi.com/2072-666X/9/1/23/s1, Figure S1: SEM images and FTIR spectra of the pristine PVA foam, PVA foam modified with SA and the Janus foam motor loaded with camphor; Figure S2: Trajectories of a typical Janus foam motor and an SA-PVA foam in 10 min; Figure S3: The contact angle of water on the surface of the SA end of the Janus foam motor; Figure S4: The oil/water separation by the Janus foam motor; Video S1: Self-propulsion of a Janus foam motor on water; Video S2: On-the-fly Oil capture and absorption by a Janus foam motor; Video S3: Oil capture and absorption by a Janus foam motor and an immobile SA-PVA foam; Video S4: Self-assembly of Janus foam motors; Video S5: Self-assembly process of two SA-PVA foams before and after oil absorption.
