# Supplementary material for: Hydrophobic Janus Foam Motors: Self-Propulsion and On-The-Fly Oil Absorption
Source: Micromachines (Basel). 2018 Jan 11;9(1):23. doi: 10.3390/mi9010023 (PMC6187715; doi:10.3390/mi9010023)
Supplement: Supplementary file 1 [file micromachines-09-00023-s001.zip › micromachines-259937-supplementary/micromachines-259937-supplementary.pdf]

micromachines

Hydrophobic Janus Foam Motors: Self-Propulsion and On-The-Fly Oil Absorption

Xiaofeng Li, Fangzhi Mou *, Jingjing Guo, Zhuoyi Deng, Chuanrui Chen, Leilei Xu, Ming Luo and Jianguo Guan *

State Key Laboratory of Advanced Technology for Materials Synthesis and Processing, International School of Materials Science and Engineering, Wuhan University of Technology, Wuhan 430070, China; xiaofegli@whut.edu.cn (X.L.); october@whut.edu.cn (J.Guo); zydeng2017@whut.edu.cn (Z.D.); chc034@ucsd.edu (C.C.); xull@whut.edu.cn (L.X.); luoming_2016@whut.edu.cn (M.L.)

* Correspondence: moufz@whut.edu.cn (F.M.); guanjg@whut.edu.cn (J.Guan)

Supporting Figures

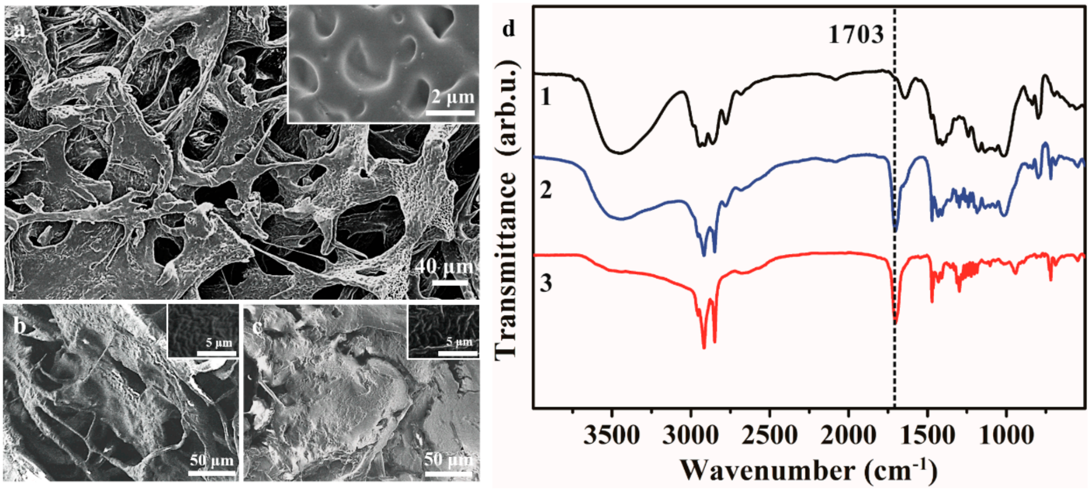

Figure S1. (**a**-**c**) SEM images and (**d**) FTIR spectra of the (**a**) pristine PVA foam, (**b**) PVA foam modified with SA, and (**c**) the Janus foam motor loaded with camphor, respectively.

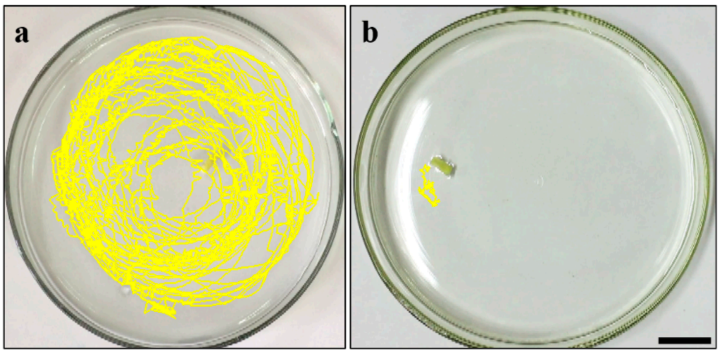

Figure S2. Trajectories of (**a**) a typical Janus foam motor and (**b**) a SA-PVA foam in 10 min. Scale bar: 20 mm.

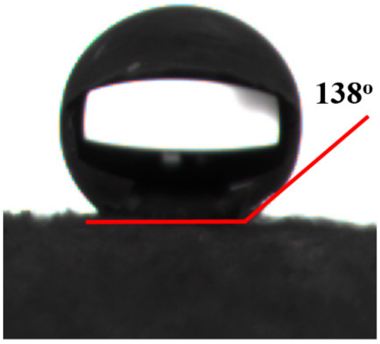

Figure S3. The contact angle of water on the surface of the SA end of the Janus foam motor.

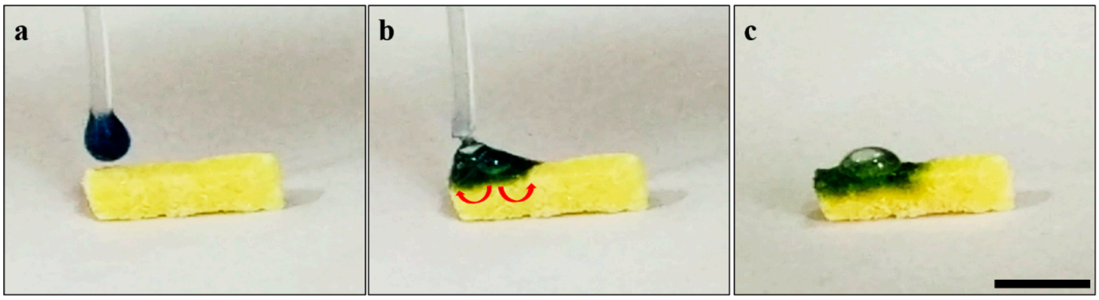

Figure S4. The oil/water separation by the Janus foam motor. When a mixed oil (colored with blue dye) and water drop (**a**) is dropped on the SA end of the motor, the oil is immediately absorbed (**b**), leaving a water droplet on the motor surface (**c**). Scale bar: 5 mm.

Supporting Videos

Supporting video 1: Self-propulsion of a Janus foam motor on water.

Supporting video 2: On-the-fly Oil capture and absorption by a Janus foam motor.

Supporting video 3: Oil capture and absorption by a Janus foam motor and an immobile SA-PVA foam.

Supporting video 4: Self-assembly of Janus foam motors.

Supporting video 5: Self-assembly process of two SA-PVA foams before and after oil absorption.
